# Clinical nurse managers’ leadership styles and staff nurses’ work engagement in Saudi Arabia: A cross-sectional study

**DOI:** 10.1371/journal.pone.0296082

**Published:** 2024-03-07

**Authors:** Amal Alluhaybi, Kim Usher, Joanne Durkin, Amanda Wilson

**Affiliations:** 1 School of Nursing and Midwifery, Faculty of Health, the University of Technology Sydney, Sydney, NSW, Australia; 2 Faculty of Nursing, Umm Al Qura University, Makkah, Saudi Arabia; 3 School of Health, University of New England, Armidale, NSW, Australia; National University of Modern Languages, PAKISTAN

## Abstract

**Background:**

Effective nurse leadership enhances nurse welfare, improves patient care, and increases organisational success. A lack of adequate, supportive leadership significantly contributes to many nurses leaving the profession. Nurse managers need to prioritise engagement and retention as significant focus areas to address the nursing shortage in Saudi Arabia and accomplish the national program’s objectives.

**Aim:**

To examine the correlation between the leadership styles of clinical nurse managers and staff engagement.

**Method:**

This study used a descriptive, cross-sectional, correlational design. The leadership styles of clinical nurse managers were evaluated using the Multifactor Leadership Questionnaire (MLQ-5X). Work engagement was assessed using the Utrecht Work Engagement Scale (UWES). Questionnaires were distributed to 450 nurses in four public hospitals in western Saudi Arabia. Non-probability convenience sampling was used to collect the data.

**Results:**

A total of 278 nurses from a range of clinical areas participated in the survey, which revealed that the leadership styles of clinical nurse managers positively or negatively impact nurse work engagement. Most clinical nurse managers exhibit transformational leadership, followed by transactional, then passive-avoidant styles. Respondents displayed a high level of work engagement, emphasising the positive impact of transformational and transactional leadership on work engagement outcomes. The findings showed significant differences in leadership styles and work engagement levels between Saudi and non-Saudi nurses across various dimensions.

**Conclusion:**

Understanding the effect of leadership styles employed by nurse managers on work engagement can positively impact staff retention rates and the quality of patient care. Nurse managers should participate in training programs to enhance their practical leadership skills to enhance the work engagement levels of nurses.

**Implication:**

Nurse work engagement can be improved by establishing training programs that promote effective leadership and highlight the significance of various leadership styles and their subsequent impact on nurse work engagement. Nursing students should receive education on leadership styles. Nursing leaders should be given access to mentoring programs and opportunities for career advancement to support the introduction of effective leadership styles.

## 1. Introduction

The healthcare sector in Saudi Arabia is undergoing significant changes as a result of the country’s Vision 2030 program, which aims to transform the nation into a self-sufficient, knowledge-driven economy [1]. One of the key objectives of this program is to train and employ Saudi nationals in different sectors to reduce the reliance on expatriates [2]. Despite this initiative, the Saudi Arabian healthcare system is experiencing a nursing shortage and continues to rely on a high percentage (57.1%) of expatriate nurses [3] to ensure continuity of patient care [4]. These nurses primarily originate from the Philippines and India, with others coming from countries including the UK, Australia, Malaysia, and Egypt. This situation has created unique challenges to providing quality care, including language barriers and cultural disparities [4, 5]. The nursing shortage significantly impacts the remaining staff, leading to increased workloads and reduced levels of high-quality, efficient patient care [4, 5]. These factors have been shown to increase secondary traumatic stress, which reduces job satisfaction and increases burnout [6, 7], resulting in a high staff turnover rate.

Addressing these challenges involves developing and implementing strategies to enhance nursing staff retention, such as enhancing nurses’ engagement in their work [8]. Work engagement refers to a positive and fulfilling mental state towards one’s work [9]. It is considered the inverse of burnout [10]. Engaged nurses are enthusiastic about their work. They feel energised and connected, enabling them to meet their job demands more effectively and making it less likely they will leave the profession [11, 12]. Work engagement has been linked to positive outcomes, including improved job performance, reduced absenteeism, staff retention and organisational success [13]. Conversely, burnout leads to exhaustion, feeling overwhelmed, and higher rates of attrition [10].

Nursing leadership plays an important role in promoting health equity, enhancing patient outcomes, and fostering a positive work environment [14]. Effective leadership significantly and positively impacts staff attitudes and behaviours [15] and may address employee-related issues [16]. Effective nursing leadership positively impacts patient outcomes, nurse satisfaction and retention, and organisational efficiency, which is essential in clinical decision-making and self-empowerment [17].

Leadership styles describe the way leaders inspire, direct, and guide their employees to achieve organisational objectives [18]. There are several categories of leadership styles, including transformational, transactional, and laissez-faire [19]. We have recently published the findings of a systematic review that showed how leadership styles affect staff engagement, with transformational leadership having a positive impact while transactional and laissez-faire leadership styles leading to negative outcomes [20]. However, the review exposed a gap in knowledge about how managerial leadership styles impact work engagement among nurses in Saudi Arabia. Understanding these dynamics is crucial for the Saudization initiative and Vision 2030 for the Saudi health system.

### 1.1 Conceptual framework

The foundation of this study is the Full Range Leadership (FRL) model proposed by Bass [21], which is the archetypal leadership paradigm for the development of leadership skills and knowledge [22, 23]. The transition from laissez-faire leadership to transactional and transformational leadership styles increases the effectiveness of processes and activities. There are four main components of transformational leadership: idealised influence (motivating followers to trust and recognise the charisma and mission of their leaders), inspirational motivation (the articulation of shared goals and the creation of a clear and convincing vision for the future that inspires followers and enhances expectations); intellectual stimulation (testing followers’ expectations, risk-taking, critical thinking, and problem-solving skills which are evident when transformational leaders present themselves as role models) [24]; and individualised considerations (recognising and understanding the developmental needs of their followers, listening to their concerns, and treating them equally) [24, 25]. Transactional leadership is contingent on reward and punishment based on employee performance [24]. Laissez-faire is the absence or avoidance of responsibility where the leader does not actively participate in leadership [25].

### 1.2 Aims, objectives, research question/hypotheses

This study aimed to study the relationship between clinical nurse manager leadership style and nurse work engagement levels in Saudi Arabia.

The objectives of the study were to:

- Determine the level of leadership style and work engagement.
- Examine the relationship between different leadership styles and work engagement.
- Identify differences in leadership styles and work engagement based on demographic variables.

### Research Question/ Hypotheses

- Q1: What is the relationship between leadership style (transformational, transactional, passive avoidant) and work engagement?
- **H0:** There is no relationship between each of the leadership styles (transformational, transactional, passive avoidant) and work engagement.
- **H1a**: There is a positive relationship between transformational leadership and work engagement.
- **H1b:** There is a negative relationship between transactional leadership and work engagement.
- **H1c**: There is a negative relationship between passive avoidant leadership and work engagement.
- Q2: Are there any differences in leadership style (transformational, transactional, passive avoidant) according to demographic variables?
- **H0**: There are no differences in leadership styles (transformational, transactional, passive avoidant) according to demographic variables.
- **H1**: Differences exist in leadership styles (transformational, transactional, passive avoidant) according to demographic variables.

## 2. Methods

### 2.1 Study design, settings, and participants

The study was a cross-sectional survey. Participants were recruited from four referral hospitals in western Saudi Arabia. At the time of data collection, these hospitals were the largest in the Makkah region. They are supported by the Saudi Ministry of Health, which oversees approximately 60% of the country’s healthcare system. A nonprobability convenience sampling technique was used to select participants. This method is commonly used in quantitative research as it is efficient, cost-effective, and convenient for researchers and participants [26]. However, convenience sampling can lead to bias as it may not represent the overall population accurately. Nurses registered with the Saudi Commission of Health Specialties, both expatriates and locals, who had worked for at least one year under their current clinical nurse managers, were eligible to participate in the English language survey. Nurses in managerial positions, such as charge nurses, head nurses, clinical directors, and clinical instructors, were excluded. Newly qualified nurses in orientation or preceptorship phases were also excluded as they were still adjusting to the work environment, and their experience may not accurately reflect that of experienced nurses.

### 2.2 Measurement instruments

The self-administered questionnaire included demographic questions and two validated instruments: the Multi-factor Leadership Questionnaire (MLQ) and the Utrecht Work Engagement Scale (UWES). Permission was obtained from the MLQ and UWES developers before using the tools. Both these instruments have been validated in Saudi Arabia [27–30].

### 2.3 Demographic and professional variables

Variables included gender, age, nationality, current employment setting, years of nursing experience, and the highest academic nursing qualification attained.

### 2.4 Multifactor leadership questionnaire (MLQ-5X)

The nurse managers’ leadership styles were analysed using the MLQ, which has forty-five items divided into twelve sub-scales. These items evaluate leadership behaviour using a five-point Likert scale (ranging from ‘not at all’ to ‘frequently, if not always’). The MLQ assesses transformational leadership by examining idealised attributes, behavioural influences, inspirational motivation, intellectual stimulation, and individual considerations. Transactional leadership is measured via contingent reward and management by exception (active), while passive avoidant behaviour is evaluated via laissez-faire and management by exception (passive).

The MLQ was psychometrically evaluated and proven dependable, with Cronbach’s coefficient alphas ranging from 0.78 to 0.94 [25, 31]. When the value is significantly more than 0.5, Cronbach’s alpha [31] indicates internal consistency.

### 2.5 Work engagement (UWES)

The Utrecht Work Engagement Scale (UWES) was employed to assess the work engagement levels of nurses. It comprises 17 items rated on a seven-point Likert scale, with responses ranging from 0 (never) to 6 (always). The instrument evaluates three sub-scales of work engagement: vigour (consisting of six items), dedication (five items), and absorption (six items). The UWES instrument demonstrated an overall Cronbach’s alpha score of 0.85 to 0.92, showing high validity and reliability [32].

### 2.6 Data collection

Data were collected online from November 2022 to January 2023 (conducted via the Qualtrics survey platform) and through hardcopies of questionnaires. The researcher (AM) held meetings with the head nurse of each unit to provide an overview of the proposed research. Participants were recruited through head nurses who disseminated an online survey link to their staff via email and WhatsApp groups (a popular social app in Saudi Arabia) and via the placement of posters and QR codes (which provided access to participant information sheets, informed consent forms, and surveys) provided by the researcher. For participants who preferred a paper copy, the principal researcher provided secure boxes in each unit for completed questionnaires. The online survey was conducted via Qualtrics using a university account and did not request identifiable information. English is the primary formal language used in the Saudi Arabian healthcare sector; therefore, the questionnaire was written in English and distributed to Saudi and non-Saudi nurses from diverse cultural backgrounds. Permission was obtained from the MLQ and UWES developers before using the tools and the participating hospitals prior to approaching head nurses.

### 2.7 Statistical analyses

The Statistical Package for Social Sciences (SPSS v.27) program was used to analyse the responses to the questionnaire. Descriptive statistics were employed to communicate the basic features of the data; continuous variables were defined using mean and standard deviation (M ± SD); and categorical variables were presented using frequency and percentage.

Inferential statistics were used to evaluate the difference between groups in the study: an independent sample T-test was employed to demonstrate the difference between the mean of two groups, specifically regarding work engagement and leadership style, and contained a variety of genders, nationalities, and work settings. One-way analysis of variance (ANOVA) tests compared results between multiple independent groups such as age and experience. Pearson’s correlation analysis was used to estimate the strength and direction of a relationship between a pair of variables. The strength of a Pearson correlation is described as low, moderate, high, and very high, depending on the numerical range. Multiple linear regression was used to model the relationship between one or more independent variables and a dependent variable; P-values of less than 0.05 were considered statistically significant.

### 2.8 Ethical considerations

Ethical approval was achieved to ensure that all relevant standards were followed. An ethics application for low-risk research was approved by the UTS (University of Technology Sydney) Human Research Ethics Committee (Project ID (ETH22-7268) as well as the Saudi Ministry of Health (IPR number IPR number H-02-K-076-07 22–775). This study followed ethical guidelines and obtaining informed consent from all participants. Those who filled out paper surveys provided written consent, while those who used the online version clicked an "Agree" button after being presented with detailed study information. The surveys were completely anonymous, with no identifying information collected.

## 3. Results

### 3.1 Response rate

Approximately 1000 nurses in total worked in the four different hospitals. Of these, 450 nurses volunteered to participate in the survey, and 278 of these met the inclusion criteria and completed the survey, giving a response rate of 61.78%.

#### 3.1.1 Demographic characteristics of the participants

This study consisted of 278 participants: the majority were female (227–81.7%); more than half were non-Saudi expatriates (167–60.1%) consisting of mainly Filipinos and Indians (35–12.0%) while fewer Egyptians, Jordanians, Tunisians, Nigerians, Malaysians, Indonesians, and Sudanese participating. Most of the respondents were BSN-educated (205–73.7%), had less than six years of experience. (111–39.9%), were aged between 30 and 39 years old (176–63.3%) and were employed in in-patient units (**Table 1**).

**Table 1:** Demographic characteristics of the participants.

Variable	Frequency	Percentage (%)
**Age**		
20–29	58	20.9
30–39	176	63.3
40–49	44	15.8
**Gender**		
Male	51	18.3
Female	227	81.7
**Working setting**		
Inpatient Unit	216	77.7
Outpatient Unit	62	22.3
**Nationality**		
Saudi	111	39.9
Non-Saudi	167	60.1
**Highest level of nursing education**		
Diploma	49	17.6
Bachelor’s degree	205	73.7
Master’s degree	24	8.6
**Total years of experience in current hospital**		
1–5 years	111	39.9
6–10 years	87	31.3
11 years and more	80	28.8

### 3.2 Mean intervals of the Likert scale

According to Pimentel [33], the five-point Likert scale has an equal interval of 4/5 (0.80) since the first interval corresponding to scale (1) is [1: 1:80] up to the fifth closed interval [4.20: 5], which corresponds to scale (5). The six-point Likert scale has an equal interval of 5/6 (0.83); the first interval is [1:1:83] corresponding to scale (1) up to the sixth closed interval [5.17: 6.00], which corresponds to scale (6). The following table shows all the mean intervals of the five- and six-point Likert scales used in the study (**Table 2**).

**Table 2:** Mean intervals of the Likert scale.

Description	Scale	Interval Length	Mean Interval
**5-Point Likert Scale**			
Not at all	1	0.80	[1: 1.80]
Once in a while	2	0.80	[1.80: 2.60]
Sometimes	3	0.80	[2.60: 3.40]
Fairly often	4	0.80	[3.40: 4.20]
Frequently, if not always	5	0.80	[4.20: 5.00]
**6-Point Likert Scale**			
Almost Never (a few times a year or less)	1	0.83	[1: 1.83]
Rarely (once a month or less)	2	0.83	[1.83: 2.67]
Sometimes (a few times a month)	3	0.83	[2.67: 3.50]
Often (once a week, about half the time)	4	0.83	[3.50: 4.33]
Very often (a few times a week)	5	0.83	[4.33: 5.17]
Always (every day)	6	0.83	[5.17: 6.00]

### 3.3 Leadership style and work engagement level

Table 3 shows transformational leadership had a mean ± SD (3.37 ± 0.70) at the interval 2.60 to less than 3.4, corresponding to scale 3 (sometimes), representing a medium level. Transactional leadership had a mean ± SD (3.30 ± .71) at the interval 2.60 to less than 3.40, corresponding to scale 3 (sometimes) representing a medium level. Passive avoidant leadership had the lowest mean score (2.55 ± 0.90), at the interval 1.80 to less than 2.60, corresponding to scale 2 (once in a while) and representing a low level.

**Table 3:** Mean and Standard deviation of leadership style and work engagement.

Variables	Mean	Std. Deviation	Level
Transformational Leadership	3.37	0.70	Sometimes
Transactional Leadership	3.30	0.71	Sometimes
Passive Avoidant	2.55	0.90	Once in a while
Vigor	3.92	1.06	Often
Dedication	4.23	1.24	Often
Absorption	3.94	1.04	Often

Regarding work engagement, the results revealed that vigour had a mean ±s (3.92± 1.06) out of 6 points at the interval 3.50 to less than 4.33, corresponding to scale 4 (often) representing a high level. Dedication had the highest mean score (4.23± 1.24), signifying a high level. The mean score of absorption (3.94± 1.04) indicated a high level. The overall mean score of work engagement had a high value (4.03± 1.05) out of 6 points, corresponding to scale 4 (often,) which represents a high level **(Table 3).**

### 3.4 Average score for the perception of leadership behaviours and work engagement outcome

According to the participants surveyed, the most common nurse manager leadership style was transformational (mean = 3.37; SD = 0.70), with inspirational motivation being the most prevalent aspect (mean = 3.37; SD = 0.70). The second most common leadership style was transactional (M = 3.30; SD = 0.71). The least common leadership style was passive avoidant (M = 2.55; SD = 0.90) **(Table 4).**

**Table 4:** Level of leadership style and work engagement among nurses in Saudi Arabia.

Variables	Mean	Std. Deviation
**Transformational Leadership**	3.37	0.70
• Idealised attributes	3.36	0.77
• Idealised behaviours	3.40	0.75
• Inspirational motivation	3.45	0.83
• Intellectual stimulation	3.32	0.76
• Individualised consideration	3.34	0.80
**Transactional Leadership**	3.30	0.71
• Contingent reward	3.39	0.80
• Management-by-exception (active)	3.21	0.84
**Passive Avoidant**	2.55	0.90
• Management-by-exception (passive)	2.62	0.92
• Laissez-faire	2.47	1.00
**Work engagement**	4.03	1.05
• Vigor	3.92	1.07
• Dedication	4.23	1.24
• Absorption	3.94	1.04

The dedication subscale of work engagement was the most frequently reported (M = 4.23; SD = 1.24), followed by absorption (M = 3.94; SD = 1.04). The vigour subscale of work engagement was the least frequently reported (M = 3.92; SD = 1.06) **(Table 4).**

### 3.5 The relationship between leadership style (transformational, transactional, passive avoidant) and work engagement

Pearson tests highlighted the following relationships: There was a significant positive correlation between transformational leadership and work engagement, where r = 0.65, p<0.01. Similarly, there was a significant positive correlation between transactional leadership and work engagement, where r = 0.56, p<0.01. In contrast, there was a significant negative correlation between passive avoidant leadership and work engagement, where r = -0.12, p<0.05. There is a strong positive correlation between transformational leadership and transactional leadership, where r = 0.828, p <0.01. The high correlation between transformational and transactional leadership styles likely reflects their complementary nature. Leaders often combine inspirational and reward-based strategies to engage and motivate teams effectively [34, 35]. (**Table 5**). Using Cohen’s guidelines, the correlation of *r* = 0.65 between transformational leadership and work engagement and *r* = 0.56 between transactional leadership and work engagement represents large effect sizes.

**Table 5:** Correlation matrix between leadership style and work engagement among nurses in Saudi Arabia.

	Work engagement	Transformational Leadership	Transactional Leadership	Passive Avoidant
Work engagement	1	.650**	.560**	-.127*
Transformational Leadership		1	.828**	.032
Transactional Leadership			1	.178**
Passive Avoidant				1

Note

*p < .05.

**p < .01.

### 3.6 Differences in leadership style (transformational, transactional, passive Avoidant) across demographic variables

Independent T-tests were performed to compare two groups in categories including gender, nationality, and work setting, while ANOVA tests were performed for age and experience groups. The results indicated a significant difference in transformational leadership style between Saudi and non-Saudi nurses (p <0.05), with non-Saudis having a higher mean score (3.45±0.68) when compared to the Saudi results (3.26±0.72). The transformational leadership style showed no significant differences when analysing the remaining demographic variables (p >0.05) (**Tables 6 and 7**).

**Table 6:** Leadership scores according to demographic variables using T-Test.

Leadership Type	Demographic	Groups	N	Mean	Std. Deviation	T-value	p-value
Transformational	Gender	Male	51	3.34	0.81	-0.38	0.71
		Female	227	3.38	0.67		
	Nationality	Saudi	111	3.26	0.72	-2.14	0.033*
		Non-Saudi	167	3.45	0.68		
	Work setting	Inpatient	216	3.38	0.70	0.07	0.94
		Outpatient	62	3.37	0.70		
Transactional	Gender	Male	51	3.32	0.83	0.21	0.84
		Female	227	3.30	0.68		
	Nationality	Saudi	111	3.21	0.71	-1.73	0.09
		Non-Saudi	167	3.36	0.70		
	Work setting	Inpatient	216	3.32	0.70	1.09	0.28
		Outpatient	62	3.21	0.73		
Passive Avoidant	Gender	Male	51	2.51	0.89	-0.31	0.76
		Female	227	2.55	0.90		
	Nationality	Saudi	111	2.74	0.80	3.07	0.002**
		Non-Saudi	167	2.42	0.94		
	Work setting	Inpatient	216	2.54	0.88	-0.07	0.94
		Outpatient	62	2.55	0.97		

Note

*p < .05.

**p < .01.

**Table 7:** Leadership scores according to age and experience using F-ANOVA.

Leadership Type	Demographic	Groups	N	Mean	Std. Deviation	F-value	p-value
**Transformational**	Age	20–29	58	3.47	0.73	0.71	0.495
		30–39	176	3.35	0.71		
		40–49	44	3.36	0.60		
	Experience	1–5 year	111	3.39	0.72	0.17	0.84
		6–10 years	87	3.39	0.68		
		11 years and more	80	3.34	0.69		
**Transactional**	Age	20–29	58	3.45	0.69	1.73	0.18
		30–39	176	3.26	0.70		
		40–49	44	3.27	0.74		
	Experience	1–5 year	111	3.34	0.71	0.33	0.72
		6–10 years	87	3.29	0.68		
		11 years and more	80	3.25	0.73		
**Passive Avoidant**	Age	20–29	58	2.52	0.91	1.36	0.26
		30–39	176	2.50	0.90		
		40–49	44	2.75	0.88		
	Experience	1–5 year	111	2.45	0.84	2.44	0.09
		6–10 years	87	2.50	0.98		
		11 years and more	80	2.72	0.87		

The analysis of transactional leadership revealed a notable difference between the results of Saudi and non-Saudi nurses (p<0.05). The non-Saudi outcomes had a higher mean score (3.36±0.70) when compared with the Saudi results (3.21±0.71). The transactional leadership style showed no significant differences when analysing the other demographic variables (p >0.05) (**Tables 6 and 7**).

Passive avoidant leadership revealed a significant difference between Saudi and non-Saudi nurses (p <0.05). Saudi nurses had a higher mean score (2.74±0.80) when compared with the outcomes for non-Saudi (2.42±0.94). According to an analysis of the remaining demographic variables, there were no other significant differences concerning the passive avoidant leadership style (p >0.05) (**Tables 6 and 7**).

### 3.7 Work engagement levels across professional demographic variables

Tables 8 and 9 shows significant differences in levels of work engagement between Saudi and non-Saudi nurses (p <0.05) with non-Saudi nurses having a higher mean score (4.23±1.00) when compared to Saudi nurses (3.73±1.07). There were no significant differences in work engagement according to the other demographic variables (p >0.05) (**Tables 8 and 9)**

**Table 8:** Results of T-Test for work engagement based on demographic variables.

Demographic	Groups	N	Mean	Std. Deviation	T-Test	p-value
**Gender**	Male	51	3.97	1.13	-0.50	0.62
	Female	227	4.05	1.04		
**Nationality**	Saudi	111	3.73	1.07	-4.00	0.00**
	Non-Saudi	167	4.23	1.00		
**Work Setting**	Inpatient Unit	216	4.01	1.08	-0.70	0.51
	Outpatient Unit	62	4.11	0.97		

**Table 9:** Results of ANOVA for work engagement based on demographic variables.

Demographic	Groups	N	Mean	Std. Deviation	F-ANOVA	p-value
**Age**	20–29	58	4.04	0.94	0.49	0.62
	30–39	176	4.00	1.09		
	40–49	44	4.20	1.07		
**Experience**	1–5 year	111	4.04	0.97	0.13	0.90
	6–10 years	87	3.99	1.13		
	11 years and more	80	4.70	1.09		

### 3.8 How demographic factors affect leadership style and work engagement: Age, gender, and education level

There were no significant differences in the study variables apart from nationality. Multiple regression showed that nationality was the only statistically significant factor affecting leadership style and work engagement across all demographic factors **(Tables 10–13)**.

**Table 10:** Multiple regression results for transformational leadership.

**Variables**	**B**	**Std. Error**	**Beta**	**t**	**p**
(Constant)	2.58	0.48		5.36	0.00
Gender	-0.02	0.12	-0.01	-0.14	0.89
**Nationality**	**0.21**	**0.09**	**0.15**	**2.31**	**0.02** *
Age	-0.09	0.08	-0.07	-1.03	0.30
Setting of work	0.06	0.11	0.04	0.58	0.56
Experience	-0.00	0.02	-0.01	-0.09	0.92
Education Level	-0.02	0.05	-0.02	-0.38	0.71

Dependent Variable: Transformational Leadership

*. Significant at the 0.05 level.

**Table 11:** Multiple regression results for transactional leadership.

Variables	B	Std. Error	Beta	t	p
**(Constant)**	3.08	0.49		6.36	0.00
**Gender**	-0.10	0.12	-0.05	-0.83	0.41
**Nationality**	0.19	0.09	0.13	2.06	0.04*
**Age**	-0.12	0.08	-0.10	-1.42	0.16
**Setting of work**	-0.06	0.11	-0.04	-0.57	0.57
**Experience**	-0.01	0.02	-0.02	-0.23	0.82
**Education Level**	-0.06	0.05	-0.07	-1.13	0.26

Dependent Variable: Transactional Leadership

*. Significant at the 0.05 level.

**Table 12:** Multiple regression results for passive avoidant.

Variables	B	Std. Error	Beta	t	p
**(Constant)**	3.83	0.61		6.32	0.00
**Gender**	0.26	0.15	0.11	1.74	0.08
**Nationality**	-0.36	0.12	-0.20	-3.09	0.00**
**Age**	0.06	0.11	0.031	0.44	0.66
**Setting of work**	-0.09	0.13	-0.04	-0.66	0.51
**Experience**	0.05	0.03	0.13	1.76	0.08
**Education Level**	-0.09	0.07	-0.081	-1.29	0.20

Dependent Variable: Passive Avoidant

**. Significant at the 0.01 level.

**Table 13:** Multiple regression results for work engagement.

Variables	B	Std. Error	Beta	t	p
**(Constant)**	1.27	0.71		1.791	0.07
**Gender**	-0.03	0.17	-0.01	-0.19	0.85
**Nationality**	0.56	0.14	0.26	4.08	0.00**
**Age**	-0.02	0.12	-0.01	-0.18	0.86
**Setting of work**	0.24	0.16	0.09	1.53	0.13
**Experience**	0.01	0.04	0.01	0.19	0.85
**Education Level**	0.00	0.08	0.00	-0.01	1.00

## 4 Discussion

This research was conducted in Saudi Arabia to investigate the leadership styles adopted by clinical nurse managers and determine how this affected nurse work engagement. The results show that staff nurses perceive their clinical managers as primarily transformational leaders, evidenced by the high scores assigned to the ’inspiration and motivation’ subscale. There was a strong correlation between this leadership style and participants’ level of engagement at work. These results are supported by previous findings that relational-oriented leadership, which emphasises the development of professional relationships with staff and maintaining high levels of interaction and trust, is one of the most effective leadership styles in health services [36]. This approach positively influences nursing practice outcomes such as job satisfaction and intention to stay in the profession [37–39], promotes quality care by improving the patient experience [40], and results in superior healthcare service satisfaction [38]. Nurse leaders collaborating with their organisations to deliver high-quality healthcare services, often adopt a transformational leadership style [41].

There is a growing demand, particularly among the younger generation, to modify the existing work environment from task-oriented leadership to a transformational leadership approach [42, 43] which includes transparency, empathy, and effective communication with nurses [42]. Leadership that provides a meaningful and inspiring vision is crucial in motivating employees to work effectively, even with a substantial workload. Incompetent management, which lacks accountability and has a poor attitude [44], can hinder the professional growth of nurses [44, 45], lead to counterproductive work practices, and create an environment that facilitates workplace bullying [46], resulting in reduced patient outcomes and satisfaction [47].

Although having transformative leaders with a relational-oriented leadership style is essential, this study found that transactional leadership combined with a task-oriented focus also received high scores from staff nurses and positively correlated with work engagement (albeit less successfully than transformational leadership). This finding is consistent with some studies but conflicts with others. Previous research suggests that transactional leadership, which focuses on ‘getting the job done’ and uses contingent rewards to motivate staff, such as bonuses or salary increases, can enhance nurses’ motivation and performance [48]. Leaders can exhibit both transformational and transactional leadership styles depending on the working environment [30, 49, 50]. However, conflicting findings indicate a shift towards transformational rather than task-oriented leadership in nursing. Transformational leaders prioritise the growth and development of their team members, while transactional leaders rely on an exchange-based relationship, which can lead to reduced satisfaction levels and negative outcomes for nurses, patients, and organisations [51]. Therefore, it is important to consider the context and conditions in which leadership is practised to understand its effectiveness. Leaders are influenced by their working environment and must adapt their behaviours and actions accordingly [52, 53]. Contextual influences, such as the leader’s presence on-site or the number of nurses in the clinic, can impact nurses’ perceptions of leadership [54]. Future research should address this knowledge gap by investigating the underlying reasons for these differences and developing an improved understanding of the unique needs of nurses and the function of nurse manager leadership in Saudi Arabia.

The results of this study revealed a significant difference between Saudi and non-Saudi nurses and their perception of transformational leadership with non-Saudis having a higher mean score (3.45±0.68) than Saudi nurses (3.26±0.72). However, there were no other significant differences in transformational leadership style based on demographic variables such as age, gender, years of experience, or education level. This suggests that the influence of cultural perspectives on leadership styles may be stronger than other demographic factors. This finding contrasts with a study conducted in Saudi Arabia, where male nurses perceived a greater utilisation of transformational leadership style than their female counterparts [28]. This discrepancy could be due to the evolving dynamics and changes in nursing management practices. Other studies have found that factors such as age, level of education, and experience can impact how staff perceive their manager’s leadership style [28, 50]. Nurses with greater employment experience or higher education levels may have different expectations and inclinations towards certain leadership styles, resulting in different perceptions of a manager’s effectiveness.

These findings highlight the complexity of leadership in nursing and emphasise the need for further research to explore the interaction between cultural, demographic, and experiential factors in shaping nurse leadership in Saudi Arabia. Understanding these dynamics can contribute to developing tailored leadership development programs and strategies that effectively support and empower Saudi nurses.

The participants in this study exhibited an elevated level of work engagement, with dedication receiving the highest score, followed by absorption and vigour. These findings align with previous studies conducted in Spain [55, 56] and the United States [57], which reported high to very high levels of work engagement among nurses. The workplace environment significantly influences nurses’ work engagement in conjunction with motivators, incentives, and autonomy in decision-making [56, 57]. Nurses who demonstrate commitment to their roles and display high energy and absorption in their work are likely to experience greater self-realisation and perceive their work as meaningful [55, 56].

This study found no statistically significant associations between work engagement and other demographic variables. However, there was a positive association between non-Saudi nurses and the level of work engagement: non-Saudi nurses displayed higher scores for work engagement when compared to their Saudi counterparts. The lower work engagement levels displayed by Saudi nurses may be attributed to the number of recent graduates with limited work experience with added pressures related to starting a family and advancing their careers [58].

This study’s findings differ from other studies conducted in Saudi Arabia, which have demonstrated that work engagement levels tend to increase with age, nursing experience, and level of education [29]. There is a significant association between work engagement and factors such as age, nursing experience, marital status, children, job position, and workplace [59]. Nurse leaders with more than 20 years of experience had higher work engagement levels than those with five years of experience or less [60]. These differences could be attributed to more years of employment, allowing nurses to explore various aspects of their profession, master their roles, and acquire knowledge [60]. Moreover, nurses with greater experience are likely to have participated in numerous training programs and workshops, enhancing their competence and performance, and positively impacting their work engagement [61].

## 5 Limitation

There are some limitations to this study that need to be considered. We used convenience sampling rather than random sampling, which may have influenced the accuracy of our sample. Our study’s design is cross-sectional, meaning we cannot draw definite cause-and-effect relationships between the study variables. Our research was conducted in only one region, so it may not apply to other contexts in Saudi Arabia. We did not collect demographic details, such as the age, nationality, and gender of nurse managers and whether they had received any leadership training. Another potential limitation is that the self-report survey is associated with the possibility of social desirability bias and memory bias, which can result in participants, consciously or subconsciously, providing inaccurate data about their experiences. To address the method bias, we performed the Harman Single factor analysis. The total variance extracted by one factor is 35%, indicating no method bias. Further qualitative research involving Saudi and non-Saudi clinical nurses could provide more profound insights into the factors shaping their experiences and opinions.

## 6. Implication

The findings of this research can aid policymakers in designing effective strategies to attract, develop, and retain Saudi nurses and reduce dependence on expatriate nurses. It can assist in reducing staff turnover rates and improve satisfaction levels among non-Saudi nurses to meet the targets identified by Vision 2030. The study clarifies the impact of leadership styles and nurse engagement while contributing to the limited literature on nursing management. It provides a platform for further research concerning the interlinking factors influencing nurse management in Saudi Arabia.

## 7. Recommendations

The study findings can be implemented to enhance nurses’ leadership and augment their work engagement in Saudi Arabia. This could be accomplished by instituting targeted training programs, creating supportive work environments, encouraging effective communication, and formulating policies acknowledging the importance of transformative leadership as a way to enhance nurse engagement.

## 8. Conclusion

The way nursing leaders approach their role significantly impacts how engaged the nurses working with them are, particularly considering changing regulations and an increased focus on work-life balance. Our research shows that transformative leadership is the preferred style. This is important as Saudi Arabia prepares for further healthcare reforms. This leadership style positively impacts work engagement, making it crucial for achieving the healthcare goals outlined in Vision 2030. Further qualitative research is needed to confirm these findings and develop effective management strategies for nursing in the Kingdom.
